# Repeated Mini-CEX encounters as a workplace-based learning tool for final-year dental students: a convergent mixed-methods study

**DOI:** 10.1186/s12909-026-09955-5

**Published:** 2026-07-25

**Authors:** Asma Siddiqui, Qudsia Sabhi, Faiza Siddiqui, Abdul Aleem, Affan Ahmad, Sonia Ijaz Haider

**Affiliations:** 1 Karachi Medical and Dental College, Karachi Metropolitan University, Karachi, Pakistan; 2 Institute of Medical Education, Jinnah Sindh Medical University, Karachi, Pakistan; 3 Dow University of Health Sciences, Karachi, Pakistan

**Keywords:** Mini-CEX, Workplace-based assessment, Dental education, Formative assessment, Feedback, Reflective learning, Competency-based education, Mixed-methods study

## Abstract

**Background:**

Workplace-based assessment is increasingly emphasized in competency-based health professions education because it allows direct observation of learners in authentic clinical settings. The Mini-Clinical Evaluation Exercise (Mini-CEX) is widely used in medical education, but its use in undergraduate dental education, particularly in Pakistan, remains limited. This study explored changes associated with repeated Mini-CEX encounters among final-year dental students and examined students’ reflective experiences related to feedback, confidence, and learning.

**Methods:**

A convergent parallel mixed-methods study was conducted among final-year Bachelor of Dental Surgery students at Karachi Medical and Dental College, Karachi, Pakistan. Forty-one students completed two Mini-CEX encounters approximately two months apart. Quantitative data included Mini-CEX domain scores, student satisfaction scores, observation time, and feedback time. Paired differences were assessed using Wilcoxon signed-rank tests, with paired-samples t-tests performed as sensitivity analyses. Qualitative data were collected through reflective essays after the second encounter and analyzed using inductive thematic analysis. Quantitative and qualitative findings were integrated through triangulation.

**Results:**

Student satisfaction increased significantly from 5.56 ± 0.63 to 7.24 ± 0.73 (*p* < 0.001). Observation time decreased from 21.71 ± 2.40 min to 7.33 ± 0.94 min, and feedback time decreased from 14.39 ± 2.55 min to 3.48 ± 0.73 min (both *p* < 0.001). Humanistic qualities/professionalism improved significantly from 4.49 ± 1.40 to 5.07 ± 0.69 (Wilcoxon *p* = 0.019). Medical interviewing scores showed a possible modest improvement from 4.54 ± 1.38 to 4.93 ± 0.52 (Wilcoxon *p* = 0.047), although the difference was not statistically significant in the paired-samples t-test sensitivity analysis. Overall clinical competence increased descriptively from 4.61 ± 1.39 to 5.00 ± 0.63, but did not reach statistical significance. Qualitative analysis generated five themes: emotional tension as a gateway to learning, feedback as a catalyst for clinical confidence, experiential learning as a bridge between theory and practice, perceived faculty attitudes shaping student engagement, and professional growth and identity formation.

**Conclusion:**

Repeated Mini-CEX encounters were associated with improved student satisfaction, reduced observation and feedback time, significant improvement in professionalism, and a possible modest improvement in medical interviewing scores. However, no statistically significant improvement was observed in overall clinical competence or in several other competency domains, including physical examination, clinical judgment, counseling skills, and organization/efficiency. The reduction in feedback time should not be interpreted as evidence of improved feedback quality. These findings suggest that repeated Mini-CEX encounters may support selected aspects of clinical learning, feedback uptake, reflective practice, and perceived professional growth, but they do not demonstrate broad improvement in overall clinical competence. Mini-CEX may be a useful formative workplace-based assessment strategy in undergraduate dental education when implemented with structured feedback, trained assessors, and appropriate monitoring of feedback quality.

**Supplementary Information:**

The online version contains supplementary material available at https://doi.org/10.1186/s12909-026-09955-5.

## Background

Competency-based health professions education has shifted the focus of assessment from knowledge recall and end-point examinations toward the demonstration of clinical competence in authentic workplace settings. In this model, learners are expected to integrate knowledge, communication, clinical reasoning, professionalism, patient-centered care, and reflective practice during real clinical encounters [1, 2]. Traditional clinical assessments, including long-case examinations and summative practical evaluations, often provide limited direct observation of learner performance and may inadequately assess how students communicate, reason clinically, organize patient encounters, and respond to feedback [3, 4]. Workplace-based assessment (WPBA) has therefore become increasingly important because it enables assessment in the clinical environment in which learners perform while also creating opportunities for feedback and guided improvement [5].

The Mini-Clinical Evaluation Exercise (Mini-CEX) is one of the most widely used WPBA tools in health professions education. It involves focused observation of a learner during a patient encounter, followed by structured feedback from an assessor. Mini-CEX is designed to assess several domains of clinical performance, including history-taking, physical examination, clinical judgment, communication, counseling, professionalism, organization, and overall clinical competence [5, 6]. Evidence from undergraduate and postgraduate medical education suggests that Mini-CEX can improve direct observation, learner engagement, feedback quality, and clinical performance when implemented through repeated encounters with trained assessors [5, 7, 8]. Its educational value has also been reported beyond medicine; for example, Motefakker et al. found that Mini-CEX-based evaluation significantly improved clinical competency among nursing students, supporting its wider relevance as a formative assessment strategy across health professions education [9].

Despite its wider use in medical education, the implementation of Mini-CEX in dental education has been slower and less extensively studied. Dental education requires students to integrate technical skills, diagnostic reasoning, communication, professionalism, and patient counseling in complex clinical settings. However, undergraduate dental assessments in many institutions remain largely summative and requirement-driven, with relatively limited structured observation and individualized feedback [10, 11]. This is important because dental students must develop not only procedural competence but also the ability to communicate effectively, respond to patient concerns, reflect on their performance, and adapt their clinical behavior. Recent studies in dental clerkship, dental radiology, orthodontics, and prosthodontics have shown that Mini-CEX can be adapted for dental training and may support learner satisfaction, formative feedback, and the assessment of clinical competence [12–15].

At the regional level, studies from Asian and low- and middle-income settings suggest that Mini-CEX and related WPBA tools are gaining attention, but their implementation remains inconsistent. Barriers include limited faculty development, time constraints in busy clinics, variability in assessor judgment, lack of standardized feedback, and student anxiety during direct observation [16–18]. These barriers may be more pronounced in undergraduate dental programs where faculty workload is high, clinical teaching time is limited, and assessment systems are often designed around summative examinations rather than longitudinal feedback. Regional studies have reported that learners value Mini-CEX for its immediate feedback, its role in building confidence, and its ability to help them identify gaps in communication and clinical performance [17–20]. However, much of the available evidence remains medical rather than dental, quantitative rather than mixedmethods, and focused on performance scores rather than learners’ reflective experiences.

In Pakistan, competency-based and feedback-oriented assessment approaches are increasingly discussed in health professions education, but their use in undergraduate dental education remains limited. Published evidence specifically documenting the implementation of structured workplace-based assessment tools in undergraduate dental programs in Pakistan is scarce. Therefore, some observations regarding current assessment practices are based on the available regional literature and the authors’ institutional experience within the local dental education context. Local dental training commonly emphasizes clinical case completion and final practical examinations, while structured observation of student-patient interactions and formal feedback may be less consistently embedded in routine clinical teaching. This observation is context-specific and should not be interpreted as nationally representative. This creates a gap between what dental curricula aim to produce—clinically competent, reflective, patient-centered graduates—and how students are often assessed during training. There is therefore a need for context-specific evidence on whether repeated Mini-CEX encounters can enhance student satisfaction, support the development of clinical competencies, and promote reflective learning among undergraduate dental students in Pakistan.

The present study was conducted among final-year Bachelor of Dental Surgery students at Karachi Medical and Dental College as part of a master’s project at Dow University of Health Sciences. The study used a mixed-methods design to evaluate two Mini-CEX encounters through quantitative assessment scores, student satisfaction ratings, observation and feedback times, and qualitative reflective essays. This design was appropriate because numerical scores alone may not fully explain how students experience observation, feedback, emotional tension, confidence-building, and professional growth. By combining competency assessment with student reflections, this study aimed to provide a broader understanding of Mini-CEX as both an assessment tool and a learning intervention.

The problem addressed in this study was the underuse of structured, feedback-oriented WPBA tools in undergraduate dental education in Pakistan. Although Mini-CEX has shown value in medical education and emerging dental contexts, there is limited local evidence regarding its role in improving student satisfaction, feedback experiences, reflective learning, and perceived clinical competence among final-year dental students. This gap is important because final-year students are close to independent clinical practice and require assessment methods that support not only performance measurement but also feedback uptake and professional identity formation.

This study, therefore, asked: How are repeated Mini-CEX encounters associated with final-year dental students’ satisfaction, clinical competency development, feedback experiences, and reflective learning in an undergraduate dental setting? The objectives were to compare student satisfaction levels between the first and second Mini-CEX encounters, assess changes in selected Mini-CEX competency domains, explore students’ perceptions of feedback and clinical learning through reflective essays, and identify how Mini-CEX may contribute to confidence, reflective practice, and professional growth.

## Methods

### Study design

This study used a convergent parallel mixed-methods design to evaluate changes associated with repeated Mini-Clinical Evaluation Exercise (Mini-CEX) encounters among final-year undergraduate dental students. A mixed-methods approach was appropriate because the study aimed to measure changes in Mini-CEX competency scores, student satisfaction, observation time, and feedback time, while also exploring students’ experiences, reflections, and perceptions of feedback [21, 22].

The quantitative strand used a single-group repeated-measures design, in which the same students were assessed during two Mini-CEX encounters. The qualitative strand used reflective essays to explore students’ perceptions of the Mini-CEX process, including their feedback experiences, emotional responses, confidence, reflective learning, and perceived professional growth.

A convergent parallel design was selected because quantitative and qualitative data were collected from the same cohort during the Mini-CEX implementation period, analyzed separately, and then integrated during interpretation [21, 22]. This design enabled comparison ofnumerical findings with students’ reflective accounts, providing a more comprehensive understanding of Mini-CEX than either quantitative or qualitative data alone [22, 23]. The mixed-methods approach was therefore suitable for evaluating Mini-CEX as both a workplace-based assessment strategy and a formative learning process.

### Conceptual framework

**Fig. 1:**
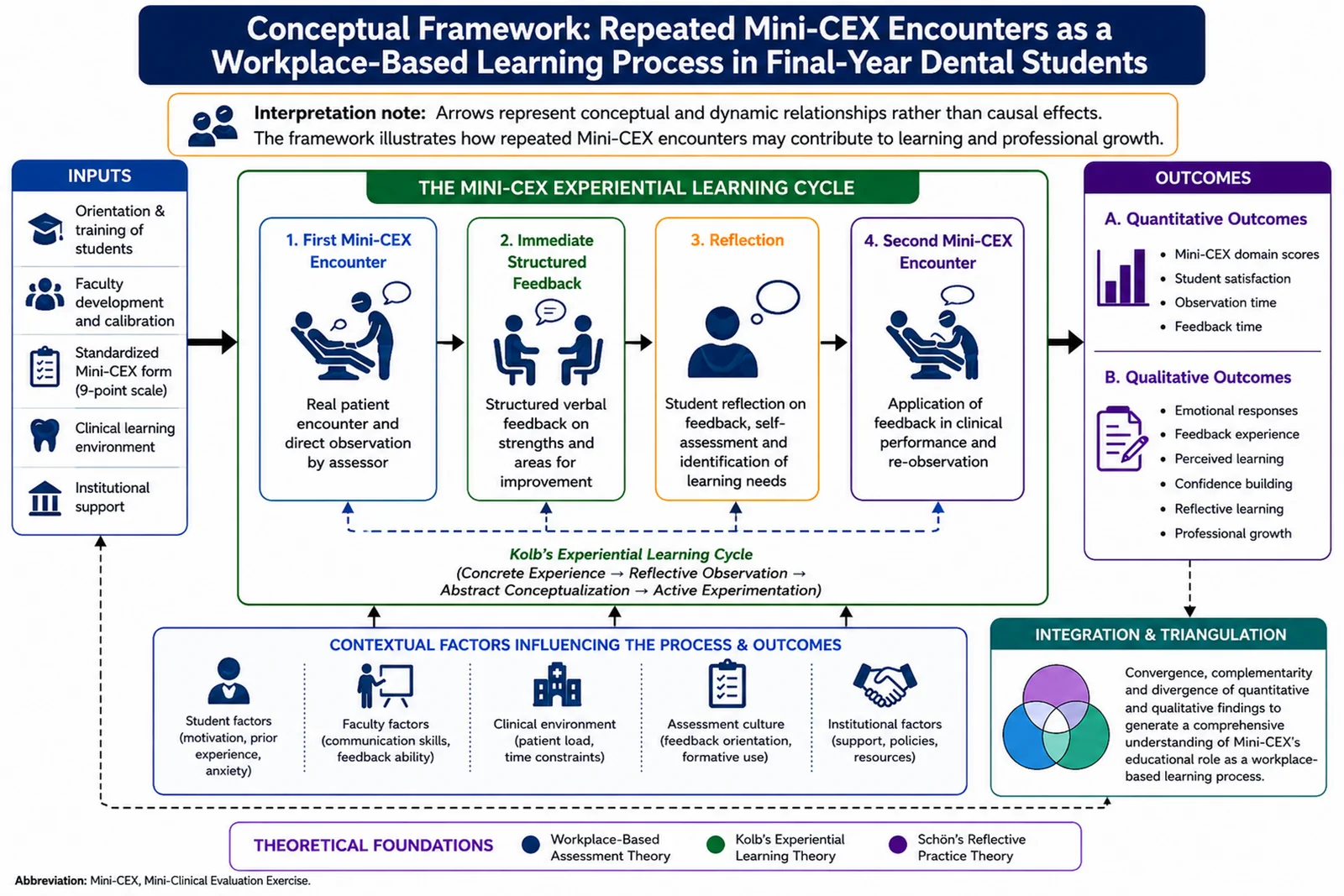
R

The conceptual framework guiding this study is shown in Fig. 1. It was informed by workplace-based assessment, Kolb’s experiential learning theory, and Schön’s reflective practice theory [5, 24, 25]. Mini-CEX was conceptualized as a formative workplace-based assessment in which direct observation, structured feedback, learner reflection, and repeated clinical performance formed a learning cycle. During the first Mini-CEX, students participated in a real clinical encounter and received immediate feedback from assessor. This feedback was expected to stimulate reflection, the identification of learning needs, and the subsequent application of feedback in the second encounter. This process aligns with Kolb’s experiential learning cycle, in which learners move from concrete experience to reflective observation, abstract conceptualization, and active experimentation [24]. It also aligns with Schön’s concept of reflective practice, in which learners develop professional judgment through reflection during and after clinical action [25].

The framework assumed that repeated Mini-CEX encounters could influence two categories of outcomes. The quantitative outcomes included Mini-CEX domain scores, student satisfaction, observation time, and feedback time. The qualitative outcomes included emotional responses, feedback experiences, perceived learning, confidence-building, reflective learning, and professional identity formation. These outcomes were integrated to understand how Mini-CEX functioned as both an assessment strategy and a learning tool.

The figure illustrates the proposed relationship among orientation, assessor preparation, direct observation, structured feedback, student reflection, and repeated clinical performance. The arrows represent conceptual and interpretive relationships rather than causal effects established by the study design. Quantitative outcomes included Mini-CEX scores, student satisfaction, observation time, and feedback time. Qualitative outcomes included emotional responses, feedback experiences, perceived learning, confidence-building, reflective learning, and professional growth. Quantitative and qualitative findings were integrated through triangulation to generate a comprehensive understanding of Mini-CEX as a formative assessment and learning process.

### Study setting

The study was conducted among final-year Bachelor of Dental Surgery students undergoing clinical training at Karachi Medical and Dental College (KMDC), Karachi, Pakistan. The study was part of a master’s project in Health Professions Education at Dow University of Health Sciences. Data were collected between April 2024 and June 2024.

### Study participants

The study population consisted of final-year undergraduate BDS students enrolled at KMDC who were actively participating in clinical rotations during the study period. Faculty members served solely as Mini-CEX assessors. They observed students, completed assessment forms, and provided structured feedback; they were not study participants, and their satisfaction was not assessed.

### Inclusion criteria

Students were eligible if they:

1. were enrolled in the final year of the BDS program at KMDC;
2. participated in both Mini-CEX encounters;
3. were present during the scheduled clinical assessment sessions;
4. provided written informed consent for participation and the submission of reflective essays.

### Exclusion criteria

Students were excluded if they:

1. declined to provide informed consent;
2. did not participate in both Mini-CEX encounters;
3. had incomplete Mini-CEX assessment forms;
4. withdrew from participation at any stage.

### Sample size and sampling technique

The sample size was calculated using G*Power 3.1 software, which is widely used for power analysis in behavioral, educational, and biomedical research [26, 27]. Based on a medium effect size of 0.5, a significance level of 0.05, and 80% statistical power, the minimum required sample size was 34. To account for possible incomplete responses, 41 final-year BDS students were included in the study.

Simple random sampling was used because the study population consisted of a defined and accessible group of final-year BDS students at KMDC. A complete list of eligible students was available from the academic office; therefore, each student was assigned a unique identification number, and students were selected at random. This approach gave every eligible student an equal probability of inclusion, minimized investigator selection bias, and improved the representativeness of the sample within the final-year BDS cohort.

### Mini-CEX orientation and assessor preparation

Before data collection, students attended an orientation session on workplace-based assessment and the Mini-CEX process. The session explained the purpose of Mini-CEX, the assessed competency domains, scoring criteria, expected clinical behavior, and the feedback process. Faculty assessors attended a separate orientation session to standardize the use of the Mini-CEX form, scoring anchors, observation process, and feedback delivery. Assessor preparation included discussion of the Mini-CEX domains, use of the 9-point rating scale, interpretation of scoring anchors, expectations for direct observation, and principles of structured verbal feedback. The orientation aimed to promote a shared understanding of the rating criteria and feedback expectations. However, formal inter-rater reliability testing was not conducted before implementation. Assessor preparation was included because faculty calibration and a shared understanding of rating criteria are important for improving the consistency and educational value in workplace-based assessment [28, 29].

### Mini-CEX procedure

Each student completed two Mini-CEX encounters during the study period. In each encounter, a faculty assessor directly observed the student during a real clinical encounter with a patient. During the first Mini-CEX encounter, students were observed while managing a clinical case and were scored using a structured, adapted Mini-CEX rating form. Immediately after the observation, the assessor provided structured verbal feedback. Feedback focused on the student's strengths, areas requiring improvement, and specific recommendations for subsequent clinical encounters.

The second Mini-CEX encounter was conducted approximately two months after the first using the same assessment format and scoring criteria. The interval was selected to allow students time to receive feedback, reflect on their performance, and continue their routine clinical learning before the second encounter. During this interval, students also continued their regular clinical rotations and patient care activities, which may have contributed to subsequent performance. Where feasible, assessor-student pairings were changed during the second encounter to minimize observer bias and reduce the influence of assessor familiarity. Complete assessor rotation was not possible in all cases because of clinical scheduling constraints. Immediate structured feedback was again provided after the second encounter. Following the completion of the second Mini-CEX, students submitted reflective essays describing their experiences, perceived strengths, areas for improvement, feedback experiences, and learning resulting from the Mini-CEX process.

### Data collection tools

Two data collection tools were used in this study: a structured Mini-CEX rating form and a reflective essay guide.

### Mini-CEX rating form

A structured Mini-CEX rating form was used to assess students’ clinical performance during both encounters. The form included seven competency domains: medical interviewing/history-taking skills, physical examination skills, humanistic qualities/professionalism, clinical judgment, counseling skills, organization/efficiency, and overall clinical competence. These competency domains are consistent with established Mini-CEX assessment formats and previous dental education studies in which Mini-CEX was adapted for clinical learning and assessment [13, 28, 29].

Each domain was rated on a 9-point scale, with scores of 1–3 indicated unsatisfactory performance, 4–6 indicated satisfactory performance, and 7–9 indicated superior performance. Similar 9-point rating scales have been used in Mini-CEX-based workplace assessment and dental education research [13, 28, 29]. The Mini-CEX form also recorded student satisfaction with the encounter, time spent on direct observation, and time spent providing feedback. Student satisfaction was recorded using the satisfaction item included in the Mini-CEX form after each encounter. Students indicated their satisfaction rating at the end of the Mini-CEX process. Because this rating was collected immediately following the feedback encounter, it may have been influenced by the assessment context, faculty presence, or social desirability. This possibility was considered when interpretating the satisfaction findings. These variables were collected for both the first and second Mini-CEX encounters to allow paired comparisons over time.

The Mini-CEX rating form was adapted from established Mini-CEX domains and previous studies using Mini-CEX in clinical and dental education. However, formal local psychometric validation of the adapted tool was not conducted before the study. Similarly, formal inter-rater reliability testing was not performed before implementation. These issues are acknowledged as methodological limitations of the study.

### Reflective essay guide

A structured reflective essay guide was used to collect qualitative data after the completion of the second Mini-CEX encounter. Reflective writing was selected because it enables learners to describe their perceptions, emotions, learning processes, and meaning-making following clinical experiences [11, 25]. Because students had completed two Mini-CEX encounters, reflective essays allowed them to compare their initial and subsequent experiences, describe how they responded to feedback, and identify perceived changes in confidence, clinical performance, and professional growth.

The guide included open-ended prompts exploring students’ initial impressions of Mini-CEX, preparation strategies, emotional responses during observation, perceptions of feedback, perceived skill development, faculty support, concerns regarding standardization, the overall value of Mini-CEX, and suggestions for improvement. These prompts were intended to capture students' experiences beyond numerical assessment scores and to provide explanatory qualitative data for the mixed-methods analysis.

Students were given three working days to complete their reflections. They were allowed to submit handwritten or typed essays. To encourage honest responses, students were informed that participation was voluntary, that their reflections would be anonymized before analysis, and that their responses would not affect their academic evaluation. Reflective essays were labeled using participant codes before coding and thematic analysis. The reflective essay guide used to collect qualitative data is provided as Additional file 2.

### Study variables

The independent variable was the time point of assessment, defined as the first Mini-CEX encounter and the second Mini-CEX encounter.

The quantitative outcome variables were Mini-CEX domain scores, student satisfaction scores, observation time, and feedback time. Mini-CEX domain scores included medical interviewing/history-taking skills, physical examination skills, humanistic qualities/professionalism, clinical judgment, counseling skills, organization/efficiency, and overall clinical competence. The qualitative areas of inquiry included students’ perceptions of feedback, emotional responses, confidence, clinical learning, reflective practice, faculty support, and professional growth. Themes were generated inductively from the reflective essays during analysis.

### Quantitative data analysis

Quantitative data were analyzed using IBM SPSS Statistics. Data were screened for completeness, missing values, and empty rows before analysis. One empty database row, which did not represent a participant, was removed during data cleaning. Descriptive statistics were calculated using frequencies and percentages for categorical variables and means with standard deviations for continuous or scale-based variables.

Because the same students completed both Mini-CEX encounters, paired analyses were performed. The Shapiro-Wilk test was applied to the paired difference scores between the first and second encounters to assess normality. Because paired differences were not normally distributed for several key variables, the Wilcoxon signed-rank test was selected as the primary inferential test for comparing first and second encounter scores. This non-parametric test was considered appropriate because it does not assume a normal distribution of paired differences and is suitable for paired ordinal or non-normally distributed continuous data. Paired-samples t-tests were retained as sensitivity analyses to examine whether conclusions were consistent under parametric assumptions. Where appropriate, effect sizes and confidence intervals were reported to support interpretation of the magnitude and precision of observed changes. Detailed normality testing of paired difference scores is provided in Additional file 1: Table S1.

The Wilcoxon signed-rank test was used to compare Mini-CEX domain scores, student satisfaction scores, observation time, and feedback time between the first and second encounters. For domains with no observed paired differences between the two encounters, inferential testing was not performed. A two-sided p-value of less than 0.05 was considered statistically significant.

Category-level changes were summarized descriptively by classifying each student’s performance as improved, unchanged, or declined between the first and second Mini-CEX encounters. Performance categories were based on the Mini-CEX scoring bands: unsatisfactory, satisfactory, and superior.

### Qualitative data analysis

Reflective essays were analyzed using inductive thematic analysis. The six-phase approach described by Braun and Clarke was followed: data familiarization, generation of initial codes, development of preliminary themes, review of themes, defining and naming of themes, and production of the final report [30, 31]. Although qualitative data were collected through reflective essays rather than interviews or focus groups, reporting was guided by established qualitative reporting principles. Relevant elements of COREQ were considered where applicable, including the description of the research team, reflexivity, participant selection, data collection context, coding procedures, theme development, and use of representative quotations. Items specific to interviews or focus groups did not apply to the use of reflective essays. Two researchers independently read the essays, generated initial codes, compared their coding decisions, and resolved disagreements through discussion. Related codes were grouped into subthemes and then organized into higher-order themes. Representative quotations were selected to support each theme.

### Researcher reflexivity and positionality

The primary investigator was trained as a dentist and was involved in dental education. This insider position provided contextual understanding of undergraduate dental training, clinical teaching practices, faculty-student interactions, and assessment challenges. However, it also created the potential for researcher influence, including assumptions shaped by prior teaching experience and the possibility that students might provide socially desirable responses because of the investigator’s professional identity.

The qualitative coding was conducted by two researchers with backgrounds in dentistry and health professions education. The primary investigator had insider knowledge of the institutional clinical learning environment, while the second coder contributed an independent review and interpretation of anonymized reflective essays. The researchers acknowledged that prior positive expectations regarding workplace-based assessment and Mini-CEX could influence their interpretation of student reflections.

To minimize these risks, reflexivity was maintained throughout the qualitative component of the study. The primary investigator kept reflexive notes during data collection and analysis to document assumptions, emerging interpretations, and possible influences of her professional background on coding decisions. Students were informed that participation was voluntary, that their decision to participate or decline would not affect their academic standing, and that their reflective essays would be anonymized before analysis. Participant identifiers were removed and replaced with codes before qualitative coding began.

To reduce the risk of single-researcher interpretation bias, two researchers independently reviewed and coded the reflective essays. Coding decisions, theme development, and the interpretation of findings were discussed until consensus was reached. Particular attention was given to negative, critical, or less favorable reflections to avoid selective interpretation of positive experiences. These included comments related to assessor variation, feelings of being unsupported, and less comprehensive guidance. This reflexive approach enhanced the credibility, transparency, and trustworthiness of the qualitative findings and helped ensure that students’ accounts were interpreted as learner experiences rather than solely through the researcher’s clinical or teaching perspective [30–33].

### Integration and triangulation

Quantitative and qualitative findings were integrated during interpretation. Integration is a central feature of mixed-methods research because it allows findings from different data sources to be compared, connected, and synthesized [22, 23]. In this study, triangulation was used to examine whether students’ reflective accounts confirmed, explained, expanded, or contradicted the quantitative findings [22, 23, 34].

Mini-CEX competency score changes were compared with students’ descriptions of perceived skill development, confidence, communication, professionalism, and reflective learning. Student satisfaction scores were interpreted alongside reflective comments about feedback quality, faculty behavior, and perceived learning value. Observation and feedback times were compared with students’ perceptions of feedback usefulness and efficiency. Areas of convergence, complementarity, and divergence were then identified to generate an integrated understanding of Mini-CEX as both an assessment strategy and a formative learning process.

### Trustworthiness and rigor

Several strategies were used to enhance rigor. For the quantitative strand, a structured Mini-CEX rating form was used; assessors were oriented before data collection, and the same assessment structure was applied across both encounters. For the qualitative strand, credibility was enhanced through repeated reading of reflective essays, independent coding by two researchers, consensus-based theme development, anonymization of participant responses, and the use of representative quotations [30, 31]. Reflexivity was maintained because the primary investigator was trained as a dentist and involved in dental education. Reflective notes were used to identify and bracket potential assumptions related to teaching experience, faculty-student relationships, and expectations regarding Mini-CEX. Attention was also paid to discrepant or critical comments to reduce selective interpretation of positive responses [32–34]. The integration of quantitative and qualitative data further strengthened the interpretation by allowing triangulation across multiple sources of evidence [22, 23, 34].

### Ethical considerations

Ethical approval was obtained from the Institutional Review Board of Dow University of Health Sciences (IRB No. 3305), and institutional permission was obtained from Karachi Medical and Dental College before data collection. Written informed consent was obtained from all participants. Participation was voluntary, and students were informed that refusal to participate or withdrawal from the study would not affect their academic standing. Data were anonymized, participant identifiers were removed, and confidentiality was maintained throughout the study.

## Results

The results are presented in three sections: quantitative findings from the two Mini-CEX encounters, qualitative findings from students’ reflective essays, and integrated mixed-methods findings. Quantitative and qualitative findings were first analyzed separately and subsequently integrated through triangulation to identify areas of convergence, complementarity, and divergence.

### Participant and encounter characteristics

Forty-one final-year BDS students completed both Mini-CEX encounters and were included in the analysis. One empty database row, which did not represent a participant, was removed during data cleaning. Most students were aged 21–22 years (*n* = 33, 80.5%), while eight students (19.5%) were aged 23 years. The sex distribution was relatively balanced, with 21 male students (51.2%) and 20 female students (48.8%).

Among the 41 encounters summarized in Table 1, most patients were aged 19–40 years (*n* = 26, 63.4%), followed by patients older than 40 years (*n* = 13, 31.7%) and patients aged 0–18 years (*n* = 2, 4.9%). Most encounters involved new patients (*n* = 34, 82.9%) and cases of moderate complexity (*n* = 29, 70.7%). The encounter focus was predominantly counseling (*n* = 40, 97.6%), while one encounter involved therapy (*n* = 1, 2.4%). Participant and encounter characteristics are summarized in Table 1.

**Table 1:** Participant and encounter characteristics

Variable	Category	*n* (%)
Student age	21–22 years	33 (80.5)
	23 years	8 (19.5)
Student sex	Male	21 (51.2)
	Female	20 (48.8)
Patient age	0–18 years	2 (4.9)
	19–40 years	26 (63.4)
	> 40 years	13 (31.7)
Patient status	New	34 (82.9)
	Follow-up	7 (17.1)
Case complexity	Low	9 (22.0)
	Moderate	29 (70.7)
	High	3 (7.3)
Encounter focus	Therapy	1 (2.4)
	Counseling	40 (97.6)

### Quantitative findings

#### Normality testing and choice of statistical test

The Shapiro-Wilk test showed that paired differences were not normally distributed for key variables with non-zero paired differences, including medical interviewing skills, humanistic qualities/professionalism, overall clinical competence, student satisfaction, observation time, and feedback time. Therefore, Wilcoxon signed-rank tests were used as the primary inferential tests for paired comparisons. Paired-samples t-tests were also performed as sensitivity analyses.

#### Mini-CEX competency scores

Mini-CEX domain scores showed possible improvement in selected areas between the first and second encounters. Medical interviewing skills increased from 4.54 ± 1.38 in the first encounter to 4.93 ± 0.52 in the second encounter. This change was statistically significant using the Wilcoxon signed-rank test (*p* = 0.047), although it did not reach statistical significance using the paired-samples t-test (*p* = 0.077). Given the difference between the two statistical tests and the p-value close to 0.05, this finding should be interpreted cautiously as a possible modest improvement. 

Humanistic qualities/professionalism increased from 4.49 ± 1.40 to 5.07 ± 0.69. This was statistically significant using both the Wilcoxon signed-rank test (*p* = 0.019) and the paired-samples t-test (*p* = 0.021). Overall clinical competence increased descriptively from 4.61 ± 1.39 to 5.00 ± 0.63; however, this change did not reach statistical significance using either the Wilcoxon signed-rank test (*p* = 0.081) or the paired-samples t-test (*p* = 0.118). No participant-level numerical change was observed in physical examination skills, clinical judgment, counseling skills, or organization/efficiency. The paired comparison of Mini-CEX domain scores between the first and second encounters is presented in Table 2.

**Table 2:** Verified paired comparison of Mini-CEX domain scores

Competency domain	First encounter Mean ± SD	Second encounter Mean ± SD	Mean difference	95% CI for mean difference	Wilcoxon *p*-value	Paired t-test *p*-value	Cohen’s dz
Medical interviewing skills	4.54 ± 1.38	4.93 ± 0.52	0.39	-0.04 to 0.82	0.047	0.077	0.28
Physical examination skills	4.80 ± 0.71	4.80 ± 0.71	0.00	Not applicable	Not applicable	Not applicable	Not applicable
Humanistic qualities/professionalism	4.49 ± 1.40	5.07 ± 0.69	0.59	0.09 to 1.08	0.019	0.021	0.37
Clinical judgment	4.76 ± 0.77	4.76 ± 0.77	0.00	Not applicable	Not applicable	Not applicable	Not applicable
Counseling skills	4.93 ± 0.72	4.93 ± 0.72	0.00	Not applicable	Not applicable	Not applicable	Not applicable
Organization/efficiency	4.83 ± 0.67	4.83 ± 0.67	0.00	Not applicable	Not applicable	Not applicable	Not applicable
Overall clinical competence	4.61 ± 1.39	5.00 ± 0.63	0.39	-0.10 to 0.88	0.081	0.118	0.25

The Wilcoxon signed-rank test was used as the primary inferential test because paired differences were not normally distributed. Paired-samples t-test results, 95% confidence intervals, and Cohen’s dz are shown as sensitivity analyses. Mean differences were calculated as the second-encounter value minus the first-encounter value. Inferential statistics and effect sizes were not calculated for domains in which all paired differences were zero. Differences may not correspond exactly to subtraction of the displayed means because calculations were based on unrounded values

#### Category-level performance change

Category-level analysis was conducted using the three Mini-CEX performance bands: unsatisfactory, satisfactory, and superior. Medical interviewing skills moved to a higher performance category in 13 students (31.7%), remained unchanged in 27 students (65.9%), and moved to a lower category in one student (2.4%). Humanistic qualities/professionalism moved to a higher category in 14 students (34.1%) and remained unchanged in 27 students (65.9%), with no decline. Overall clinical competence moved to a higher category in 13 students (31.7%), remained unchanged in 27 students (65.9%), and moved to a lower category in one student (2.4%). Category-level changes between the first and second Mini-CEX encounters are shown in Table 3.

No participant-level numerical or categorical change was observed in physical examination skills, clinical judgment, counseling skills, or organization/efficiency. Detailed distributions of Mini-CEX performance categories for the first and second encounters are provided in Additional file 1: Table S3.

**Table 3:** Category-level change between the first and second Mini-CEX encounters

Competency domain	Improved *n* (%)	No change *n* (%)	Declined *n* (%)
Medical interviewing skills	13 (31.7)	27 (65.9)	1 (2.4)
Physical examination skills	0 (0.0)	41 (100.0)	0 (0.0)
Humanistic qualities/professionalism	14 (34.1)	27 (65.9)	0 (0.0)
Clinical judgment	0 (0.0)	41 (100.0)	0 (0.0)
Counseling skills	0 (0.0)	41 (100.0)	0 (0.0)
Organization/efficiency	0 (0.0)	41 (100.0)	0 (0.0)
Overall clinical competence	13 (31.7)	27 (65.9)	1 (2.4)

#### Student satisfaction, observation time, and feedback time

Student satisfaction increased significantly from 5.56 ± 0.63 in the first encounter to 7.24 ± 0.73 in the second encounter. This increase was statistically significant using both the Wilcoxon signed-rank test and the paired-samples t-test (*p* < 0.001).

Observation time decreased significantly from 21.71 ± 2.40 min in the first encounter to 7.33 ± 0.94 min in the second encounter (*p* < 0.001). Feedback time also decreased significantly from 14.39 ± 2.55 min to 3.48 ± 0.73 min (*p* < 0.001).

These findings indicate that the second Mini-CEX encounter was associated with higher student satisfaction and shorter observation and feedback times. However, reduced feedback time should be interpreted cautiously. It may reflect improved procedural efficiency as students and assessors became more familiar with the Mini-CEX process, but it may also indicate reduced feedback depth, less comprehensive guidance, or variability in assessor engagement. Therefore, shorter feedback duration should not be interpreted as direct evidence of improved feedback quality. The comparison of student satisfaction, observation time, and feedback time is presented in Table 4.

**Table 4:** Verified comparison of student satisfaction and Mini-CEX timing

Variable	First encounter Mean ± SD	Second encounter Mean ± SD	Mean difference	95% CI for mean difference	Wilcoxon *p*-value	Paired t-test *p*-value	Cohen’s dz
Student satisfaction score	5.56 ± 0.63	7.24 ± 0.73	1.68	1.38 to 1.99	< 0.001	< 0.001	1.75
Observation time, minutes	21.71 ± 2.40	7.33 ± 0.94	-14.38	-15.19 to -13.56	< 0.001	< 0.001	-5.58
Feedback time, minutes	14.39 ± 2.55	3.48 ± 0.73	-10.91	-11.70 to -10.11	< 0.001	< 0.001	-4.34

Observation and feedback times are reported according to the first and second Mini-CEX encounter sequences. Wilcoxon signed-rank tests were used as the primary inferential tests. Paired-samples t-test results, 95% confidence intervals, and Cohen’s dz are shown as sensitivity analyses. Mean differences were calculated as the second-encounter value minus the first-encounter value. Negative mean differences and effect sizes indicate shorter durations during the second encounter

#### Qualitative findings

All submitted reflective essays were anonymized before analysis and labeled using participant codes. Inductive thematic analysis generated five overarching themes: emotional tension as a gateway to learning, feedback as a catalyst for clinical confidence, experiential learning as a bridge between theory and practice, perceived faculty attitudes shaping student engagement, and professional growth and identity formation.

Initial coding generated codes related to nervousness, apprehension, curiosity, preparation, feedback specificity, confidence, clinical adaptation, faculty support, and professional development. These codes were clustered into subthemes and then organized into the five overarching themes. The expanded qualitative coding framework is provided in Additional file 1: Table S4, and the reflective essay guide is provided in Additional file 2. The qualitative findings suggested that students experienced Mini-CEX as an emotionally demanding but educationally meaningful process that supported feedback uptake, confidence-building, authentic clinical learning, and professional growth.

### Theme 1: emotional tension as a gateway to learning

Students described mixed emotional responses before and during Mini-CEX encounters. Common codes included nervousness, apprehension, anxiety, fear, excitement, pressure, curiosity, and motivation. Direct observation by faculty initially created performance pressure, but students also described this emotional tension as a trigger for preparation, attention, and improvement. For several students, anxiety appeared to decrease after receiving constructive feedback.

Representative quotation:

- “I felt nervous, but the feedback made me want to improve next time.” (P13)

This theme suggests that emotional discomfort during direct observation was not only a barrier but also a potential starting point for learning when followed by supportive feedback.

### Theme 2: feedback as a catalyst for clinical confidence

Students repeatedly emphasized the importance of feedback that was specific, immediate, and actionable. Codes supporting this theme included constructive feedback, real-time feedback, targeted skill development, actionable insights, improved confidence, and boosted confidence. Students valued feedback when it identified specific areas for improvement rather than providing general comments.

Representative quotation:

- “The assessor pointed out a specific step I missed in my exam routine.” (P9)

This theme complements the quantitative improvement in satisfaction scores and suggests that feedback quality was central to students’ positive perceptions of Mini-CEX.

### Theme 3: experiential learning bridging theory and practice

Students described Mini-CEX as an authentic learning experience that connected theoretical preparation with clinical practice involving real patients. Codes included reviewing clinical guidelines, practicing clinical skills, simulation exercises, role-play, real-world scenarios, communication skills, clinical decision-making, patient safety, and hands-on learning. Students reported that Mini-CEX encouraged them to apply knowledge in practical clinical situations.

Representative quotation:

- “I had to think on my feet with a real patient; it was nothing like OSCE.” (P18)

This theme indicates that Mini-CEX helped students connect classroom learning with clinical reasoning and patient interaction.

### Theme 4: perceived faculty attitudes shaping student engagement

Students’ engagement with Mini-CEX was influenced by how assessors communicated during observation and feedback. Codes included excellent support and guidance, helpful and encouraging behavior, constructive feedback, non-judgmental attitude, supportive and kind attitude, detailed feedback, and useful guidance. Less favorable codes, including feelings of being unsupported, less comprehensive guidance, variability in assessor engagement, and disinterest, were also identified. These findings suggest that faculty behavior and feedback style influenced students’ openness to learning.

Representative quotation:

- “The teacher listened and guided without judgment. I respected that.” (P26)

This theme highlights the educational importance of maintaining psychological safety during workplace-based assessment.

### Theme 5: professional growth and identity formation

Students described Mini-CEX as more than a score-based assessment. Codes included personal development, professional competency, holistic development, resilience and growth, refined skills, professional behaviors, and profound educational influence. These codes suggest that students perceived Mini-CEX as contributing to self-awareness, responsibility, and confidence in their clinical roles.

Representative quotation:

- “I felt like a real dentist, not just a student ticking boxes.” (P5)

This theme suggests that Mini-CEX supported professional identity formation by making students more aware of their role in patient care. The qualitative themes, subthemes, codes, and representative quotations are summarized in Table 5.

**Table 5:** Qualitative themes, subthemes, codes, and representative quotations

Theme	Subthemes	Example codes	Representative quotation
Emotional tension as a gateway to learning	Anticipation; anxiety; curiosity; emotional transition	Nervousness, apprehension, anxiety, fear, excitement, pressure, curiosity, motivation	“I felt nervous, but the feedback made me want to improve next time.” (P13)
Feedback as a catalyst for clinical confidence	Specific feedback, actionable correction, confidence-building	Constructive feedback, real-time feedback, targeted skill development, actionable insights, improved confidence, boosted confidence	“The assessor pointed out a specific step I missed in my exam routine.” (P9)
Experiential learning: bridging theory and practice	Preparation, real-world learning, and application of knowledge	Reviewed clinical guidelines, practiced skills, simulation exercises, role-play, real-world scenarios, communication skills, clinical decision-making, and patient safety	“I had to think on my feet with a real patient; it was nothing like OSCE.” (P18)
Perceived faculty attitudes shaping student engagement	Supportive faculty behavior; psychological safety; assessor variation	Excellent support and guidance, helpful, encouraging, non-judgmental, constructive feedback, feelings of being unsupported, less comprehensive guidance, variation	“The teacher listened and guided without judgment. I respected that.” (P26)
Professional growth and identity formation	Personal development, professional responsibility, and holistic growth	Personal development, professional competency, resilience and growth, professional behaviors, holistic development, and profound educational influence	“I felt like a real dentist, not just a student ticking boxes.” (P5)

### Mixed-methods integration and triangulation

Quantitative and qualitative findings were integrated to examine whether students’ reflective accounts confirmed, explained, expanded, or contradicted the numerical findings. Overall, the two strands showed convergence for student satisfaction and humanistic qualities/professionalism, partial convergence for medical interviewing, and complementarity for overall clinical competence. The mixed-methods integration process is summarized in Additional file 1: Fig. S1.

The significant increase in student satisfaction was consistent with qualitative codes related to constructive feedback, real-time feedback, supportive faculty behavior, confidence-building, and perceived learning value. This suggests that higher satisfaction variability in assessor engagement of feedback as specific, timely, and educationally useful.

The significant improvement in humanistic qualities/professionalism also converged with qualitative findings related to non-judgmental faculty guidance, professional competency, personal development, and professional behaviors. This indicates that Mini-CEX may have supported students’ professional awareness, accountability, and reflective development during patient-based clinical encounters with patients.

Medical interviewing showed partial convergence. Scores showed a possible modest improvement and reached statistical significance on the Wilcoxon signed-rank test, but not on the paired-samples t-test sensitivity analysis. Qualitative findings related to communication skills, patient interaction, real-world scenarios, and confidence provided supportive evidence for communication-related learning. However, this finding should be interpreted cautiously because statistical evidence differed by test.

Overall clinical competence increased descriptively but did not reach statistical significance. Qualitative findings suggested perceived growth in confidence, clinical learning, self-reflection, and professional development. This represents complementarity rather than full statistical convergence, as students perceived broader growth while the overall competence score did not show statistically significant improvement.

The reduction in observation and feedback time was quantitatively strong. Qualitative findings suggested that students valued focused and constructive feedback but also indicated variation in guidance and feedback comprehensiveness. Therefore, reduced time may indicate improved procedural efficiency; however, future implementation should ensure that shorter feedback remains sufficiently detailed, focused, and educationally useful. The integrated quantitative and qualitative findings are presented in Table 6.

**Table 6:** Joint display of integrated quantitative and qualitative findings

Quantitative finding	Qualitative finding	Integrated interpretation
Student satisfaction increased significantly from 5.56 ± 0.63 to 7.24 ± 0.73; *p* < 0.001.	Codes included constructive feedback, real-time feedback, supportive guidance, confidence, and perceived learning value.	Strong convergence: satisfaction improved, and students described feedback as educationally valuable.
Humanistic qualities/professionalism improved significantly from 4.49 ± 1.40 to 5.07 ± 0.69; Wilcoxon *p* = 0.019.	Codes included professional competency, professional behaviors, support, guidance, and personal development.	Convergence: quantitative improvement aligned with professional growth-related reflections.
Medical interviewing showed a possible modest improvement from 4.54 ± 1.38 to 4.93 ± 0.52; Wilcoxon *p* = 0.047, paired t-test *p* = 0.077.	Codes included communication skills, real-world scenarios, clinical interaction, and confidence.	Partial convergence: qualitative findings support communication improvement, but statistical evidence was modest.
Overall clinical competence increased descriptively from 4.61 ± 1.39 to 5.00 ± 0.63, but was not statistically significant; Wilcoxon *p* = 0.081.	Codes suggested confidence, improved skills, self-reflection, and professional growth.	Complementarity: students perceived growth, but the overall score did not show statistically significant improvement.
Observation and feedback time decreased significantly; *p* < 0.001.	Codes included useful guidance and constructive feedback, but also variation and less comprehensive guidance.	Reduced time may indicate efficiency, but feedback quality should be monitored.
Category-level improvement was observed mainly in medical interviewing and professionalism, with descriptive improvement in overall clinical competence.	Codes indicated confidence-building, motivation, and reflective learning.	Category-level findings were consistent with perceived confidence-building; however, qualitative reflections did not establish improvement in domains with unchanged numerical scores.

The integrated findings demonstrated three forms of mixed-methods interpretation. First, convergence was observed for student satisfaction and professionalism, where quantitative improvement aligned with qualitative reflections describing timely feedback, supportive faculty behavior, and professional growth. Second, complementarity was observed for overall clinical competence, where students described perceived confidence and growth, but quantitative scores did not show statistically significant improvement. Third, caution was required when interpreting feedback duration, because quantitative results showed shorter feedback times while qualitative data suggested variability in feedback quality and comprehensiveness. These findings support a balanced interpretation of Mini-CEX as a potentially useful formative learning process, while also emphasizing the need to monitor feedback quality and avoid overinterpreting score changes.

### Overall summary of results

**Fig. 2:**
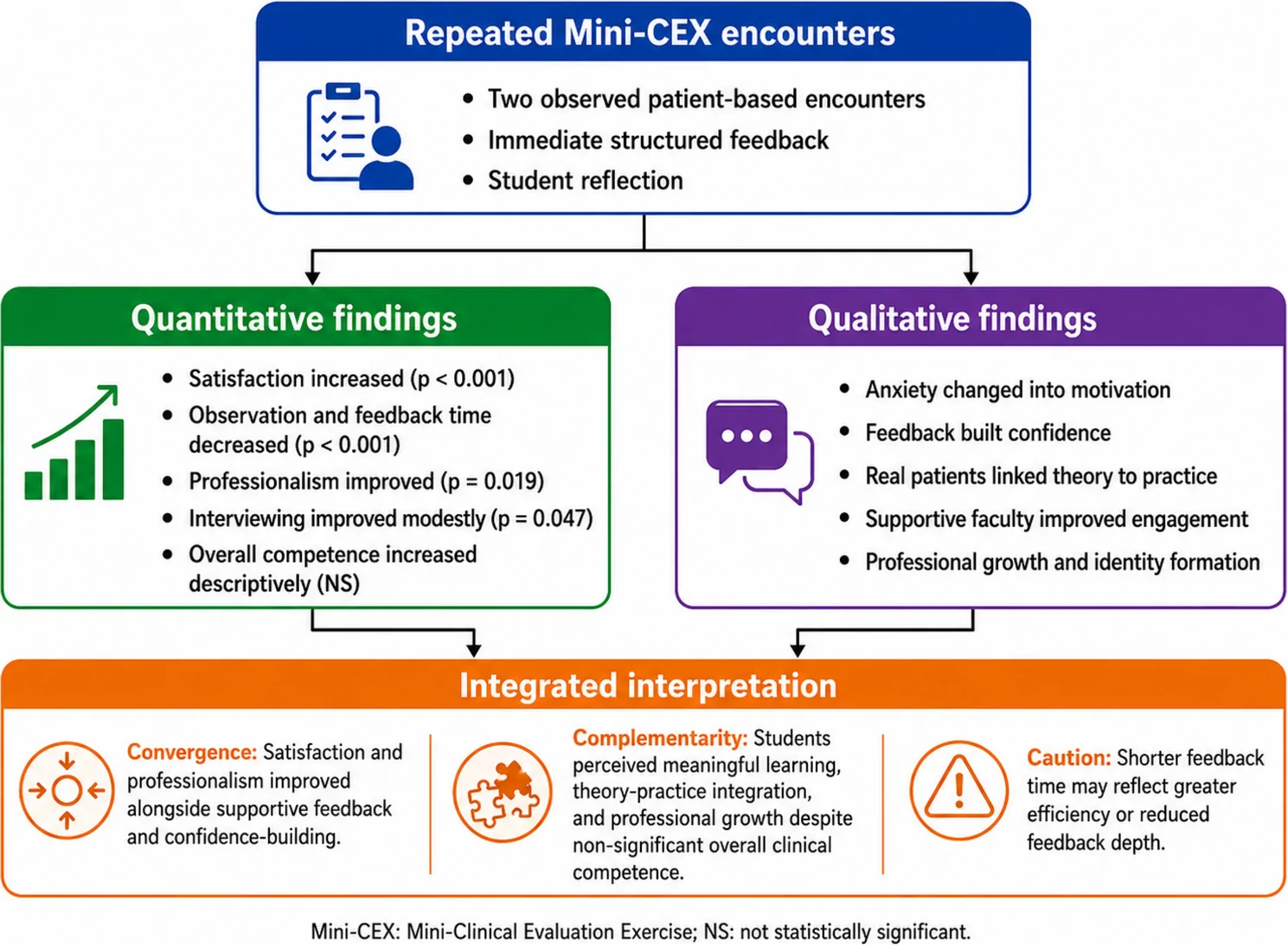
Summary of integrated findings from repeated Mini-CEX encounters

Figure 2 summarizes the quantitative findings, qualitative findings, and mixed-methods integration. Convergence was observed between increased satisfaction and students’ reflections on timely, supportive, and specific feedback. Complementarity was observed where students described confidence and professional growth despite the non-significant improvement in overall clinical competence. Caution was required when interpreting reduced feedback time because the shorter duration may reflect improved efficiency but may also indicate reduced feedback depth or variability in feedback quality.

## Discussion

This convergent mixed-methods study explored repeated Mini-CEX encounters as a workplace-based assessment and learning strategy for final-year undergraduate dental students. The main quantitative findings were increased student satisfaction, reduced observation and feedback times, a statistically significant increase in humanistic qualities/professionalism, and a possible modest improvement in medical interviewing skills. Overall clinical competence increased descriptively but did not reach statistical significance. No measurable improvement was observed in physical examination skills, clinical judgment, counseling skills, or organization/efficiency. Therefore, the findings should be interpreted as showing improvement in selected competency domains and perceived learning experiences rather than demonstrating broad improvement in overall clinical competence. The qualitative findings complemented these results by showing that students experienced Mini-CEX as a feedback-rich, emotionally engaging, and clinically authentic learning process that supported confidence, reflection, and professional growth.

The lack of measurable change in several domains may reflect limited sampling across only two encounters, restricted score variability, assessor-related effects, differences in case relevance, or limited sensitivity of the rating scale. This may be particularly relevant to physical examination, clinical judgment, counseling skills, and organization/efficiency, for which no participant-level numerical change was observed.Therefore, unchanged scores should not be interpreted as definitive evidence that Mini-CEX had no educational value in these areas. Rather, the findings may reflect insufficient sampling across encounters, assessors, clinical contexts, and case types.

The significant improvement in humanistic qualities/professionalism is an important finding because professionalism is difficult to teach and assess through conventional written or procedural examinations. Mini-CEX may have made professional behaviors more visible by placing students in directly observed clinical encounters with patients, during which communication, respect, empathy, and accountability were directly assessed. This aligns with workplace-based assessment literature, which emphasizes that authentic clinical observation is valuable for assessing competencies that are otherwise less visible in traditional summative assessments [5, 28, 29]. It also resonates with recent dental professional identity literature, which shows that clinical experiences, faculty role modeling, and reflection help students understand their responsibilities as future oral health professionals [35]. However, because the study lacked a comparison group, the observed change cannot be attributed solely to Mini-CEX.

Medical interviewing showed a possible modest improvement and reached statistical significance in the Wilcoxon analysis but not in the paired-samples t-test sensitivity analysis. This finding should therefore be interpreted cautiously. Communication and interviewing skills may develop through repeated observation, targeted feedback, and opportunities to apply feedback in subsequent encounters. The qualitative findings support this interpretation, as students described feedback as specific, actionable, and useful for improving patient interaction. This is consistent with previous studies showing that Mini-CEX can strengthen feedback uptake, learner confidence, and communication-related learning when embedded in real clinical settings [5, 7, 28, 36].

Overall clinical competence increased descriptively but did not reach statistical significance. This is understandable because overall competence is a broad construct that cannot be reliably judged on the basis of only two encounters. Van der Vleuten and Schuwirth argue that competence assessment should move beyond isolated methods toward assessment programmes that collect multiple data points across contexts, assessors, and tasks [35]. Similarly, programmatic assessment emphasizes assessment for learning, in which repeated low-stakes assessments are combined to support development and inform more credible judgments of competence [37]. Therefore, the non-significant change in overall competence may reflect limited sampling rather than the absence of educational value.

Student satisfaction increased markedly after the second Mini-CEX encounter. The qualitative findings suggested that this increase was related, at least in part, to students’ appreciation of feedback that was immediate, respectful, specific, and linked to observable performance. This indicates that satisfaction was not merely a reaction to being assessed but was connected to the perceived usefulness of the feedback process. The finding is consistent with feedback literature in dental education, which highlights that feedback is most effective when it is timely, behavior-specific, and oriented toward future improvement [10, 11, 38]. However, satisfaction should not be treated as equivalent to competence; rather, it reflects learner acceptability and perceived educational value.

Observation and feedback times decreased significantly in the second encounter. This may indicate improved process efficiency as students and assessors became more familiar with the Mini-CEX format. However, shorter feedback duration should not automatically be interpreted as evidence of better or more efficient feedback. A reduction in feedback time may also reflect reduced feedback depth, less comprehensive discussion, or variability in assessor engagement. This interpretation is supported by the qualitative findings, in which students valued focused and constructive feedback but also reported variation in guidance and feedback comprehensiveness. Therefore, future implementation should monitor feedback quality in addition to feedback duration. In busy dental clinics, this is particularly important because rushed feedback can reduce the formative value of workplace-based assessment [16, 29].

The qualitative findings added depth to the quantitative results. Students initially experienced anxiety and performance pressure, but this emotional tension appeared to become educationally productive when followed by supportive feedback. This suggests that psychological safety is central to Mini-CEX implementation. Students are more likely to engage with feedback when assessors are respectful, non-judgmental, and specific. The themes of confidence-building, theory-practice connection, and professional identity formation support the conceptual framework of this study, in which clinical experience, feedback, reflection, and repeated performance functioned as linked mechanisms of learning. These findings are consistent with Kolb’s experiential learning theory and Schön’s reflective practice, which emphasize that learning occurs when experience is followed by reflection and future action [24, 25].

The findings can be interpreted more explicitly through the theoretical frameworks underpinning this study. In relation to Kolb’s experiential learning cycle, the first Mini-CEX encounter provided a concrete clinical experience, immediate feedback supported reflective observation, students’ identification of strengths and areas for improvement represented abstract conceptualization, and the second encounter offered an opportunity for active experimentation. Schön’s reflective practice theory was reflected in students’ descriptions of becoming more aware of their clinical behavior, communication, and professional responsibilities after the encounter. The reflective essays primarily demonstrated reflection-on-action, while some student accounts also suggested reflection-in-action as learners described adapting their approach during patient interaction. From a workplace-based assessment perspective, these findings suggest that Mini-CEX may function as an assessment-for-learning strategy, in which direct observation and feedback function not only to generate scores but also to guide learning, confidence-building, and professional identity formation.

### Strengths

This study has several strengths. First, it used a convergent parallel mixed-methods design, allowing quantitative changes to be interpreted alongside students’ reflective experiences. Second, the study was conducted in a real clinical dental setting, which enhanced its ecological validity. Third, the same students completed two encounters, allowing paired within-student comparisons. Fourth, the qualitative component captured learner's experiences beyond numerical scores, especially emotional responses, feedback experiences, and professional identity formation. Finally, reflexivity and triangulation strengthened the credibility of the qualitative and mixed-methods interpretations.

This study adds to the existing Mini-CEX literature in dental education in several ways. First, it provides context-specific evidence from undergraduate dental education in Pakistan, where published evidence on structured workplace-based assessment remains limited. Second, rather than examining Mini-CEX only as an assessment format, this study explored it as a formative learning process by integrating competency scores, satisfaction, timing data, and reflective narratives. Third, the qualitative component highlighted emotional tension, feedback experiences, faculty behavior, psychological safety, and professional identity formation, offering insight into how students experienced Mini-CEX beyond numerical ratings. Finally, the mixed-methods integration clarified where quantitative and qualitative findings converged, complemented each other, or required cautious interpretation.

### Limitations

The study also has limitations. It was conducted at a single institution with a relatively small sample of final-year dental students, which limits the generalizability of the findings. Only two Mini-CEX encounters were conducted; more encounters across varied cases and assessors may be needed to detect changes in clinical competence. The case mix was heavily weighted toward counseling encounters, limiting the assessment of procedural and technical domains. Assessor variability may have influenced scores despite the orientation and attempted asrotation of assessors.

The absence of a control or comparison group is an important limitation. Therefore, the observed changes should not be interpreted as causal effects of Mini-CEX or feedback alone. During the two-month interval between encounters, students continued their routine clinical rotations and may have gained experience through additional patient care exposure, informal faculty guidance, peer learning, maturation, practice effects, reduced anxiety, and increased familiarity with the assessment process. The observed changes should therefore be interpreted as being potentially associated with the overall Mini-CEX experience and surrounding clinical learning environment, rather than as direct effects of Mini-CEX feedback alone.

The adapted Mini-CEX rating form was based on established domains, but formal local psychometric validation was not conducted before the study. Formal inter-rater reliability testing was also not performed before implementation. Although assessor orientation was provided, variability in assessors' interpretation of the rating criteria may have influenced competency scores.

Several competency domains showed no measurable change. This may reflect limited encounter sampling, restricted score variability, assessor-related effects, differences in case relevance, or limited sensitivity of the 9-point rating scale over only two encounters. Because no participant-level numerical change was observed in these domains, the findings should also be verified against the original dataset to exclude possible data-entry or coding errors.

Feedback quality was not measured using a validated feedback quality tool. Therefore, the reduction in feedback time cannot be assumed to represent improved feedback efficiency alone; it may also reflect reduced feedback depth or less comprehensive guidance in some encounters. Reflective essays may have been influenced by social desirability bias, particularly because the primary investigator was a dentist and involved in dental education. Student satisfaction ratings may also have been affected by social desirability or faculty presence because ratings were completed soon after the feedback encounter. Finally, because qualitative data were collected through reflective essays rather than interviews or focus groups, opportunities for probing or clarification of student responses were limited.

In addition, several competency domains and process outcomes were examined without adjustment for multiple comparisons. Therefore, domain-level findings, particularly p-values close to 0.05, should be interpreted as exploratory.

### Policy and educational implications

The findings suggest potential value in the structured integration of Mini-CEX into undergraduate dental curricula as a low-stakes formative assessment strategy. Dental institutions may consider moving beyond requirement-based case completion and include direct observation, structured feedback, and learner reflection as routine parts of clinical training. Mini-CEX should not be used as a stand-alone high-stakes measure of competence. Instead, it should contribute to a broader system of repeated observations across different cases, assessors, and time points. However, Mini-CEX should be implemented as part of a broader programmatic assessment approach rather than as a stand-alone measure of competence. The present findings support its potential formative value, but they do not establish that two Mini-CEX encounters are sufficient to determine overall clinical competence. Multiple encounters across different clinical tasks, assessors, and settings are required for more credible judgments of learner development.

At the institutional level, successful implementation requires structured and contextually appropriate Mini-CEX forms, faculty development, assessor calibration, protected feedback time, and monitoring of feedback quality. Faculty training is particularly important because the educational value of Mini-CEX depends heavily on assessor behavior, feedback quality, and psychological safety. At the policy level, dental regulatory and curriculum bodies in Pakistan may consider supporting the careful integration and evaluation of workplace-based assessment within competency-based dental education and helping institutions in develop feasible, locally adaptable implementation models.

### Way forward

Future research should evaluate Mini-CEX across multiple dental institutions and larger student cohorts. Studies should include more than two encounters, varied case types, multiple assessors, and longer follow-up to determine whether improvements are sustained. Future work should also incorporate objective measures of feedback quality, assessor training outcomes, and downstream performance indicators such as OSCE scores, clinical examination performance, or readiness for internship. Digital Mini-CEX platforms may help standardize documentation, track longitudinal progress, and support programmatic assessment. Further qualitative work should explore faculty perspectives, barriers to implementation, and how students use feedback to change future clinical behavior. Future studies should also include control or comparison groups where feasible to help distinguish Mini-CEX-related learning from maturation, routine clinical exposure, practice effects, and increased familiarity with assessment. Further research should formally evaluate the validity and reliability of adapted Mini-CEX tools in undergraduate dental contexts and include validated measures of feedback quality.

## Conclusion

Repeated Mini-CEX encounters were associated with improved student satisfaction, reduced observation and feedback times, a statistically significant increase in humanistic qualities/professionalism, and a possible modest improvement in medical interviewing skills. However, no statistically significant improvement was observed in overall clinical competence, and no measurable change was found in physical examination, clinical judgment, counseling skills, or organization/efficiency. Therefore, the findings support improvement in selected competency domains and perceived learning experiences rather than broad improvement in overall clinical competence. The reduction in feedback time should not be interpreted as evidence of improved feedback quality.

Qualitative findings showed that students perceived Mini-CEX as useful for feedback uptake, confidence-building, reflective learning, theory-practice integration, and professional identity formation. These qualitative findings complemented the quantitative results but should not be interpreted as proof of causal educational effectiveness. In the absence of a control group, the observed changes may reflect multiple interacting influences, including repeated clinical exposure, maturation, practice effects, reduced anxiety, and familiarity with the Mini-CEX process.

When implemented with trained assessors, structured feedback, protected time, and attention to feedback quality, Mini-CEX may serve as a useful formative workplace-based assessment strategy in undergraduate dental education. Further controlled, longitudinal, multi-institutional studies using validated tools, multiple assessors, diverse case types, and objective measures of feedback quality are needed before stronger conclusions can be drawn regarding its impact on overall clinical competence.

## Supplementary Information

Additional file 1. Supplementary supporting analyses and mixed-methods integration figure. This file includes Table S1, normality testing of paired difference scores; Table S2, extended paired comparison results and sensitivity analyses; Table S3, distribution of Mini-CEX performance categories in the first and second encounters; Table S4, expanded qualitative coding framework; and Fig. S1, mixed-methods integration process.

Additional file 2. Qualitative reflective essay guide. This file includes the reflective essay guide used to collect qualitative data after the second Mini-CEX encounter, which features open-ended prompts and aligns with the qualitative themes reported in the manuscript.

Additional file 3.

## Data Availability

The datasets generated and/or analyzed during the current study are not publicly available because they contain student assessment data and reflective responses from a single educational institution. De-identified data may be made available from the corresponding author on reasonable request and with permission from the relevant institutional authorities.
