# Supplementary material for: Repeated Mini-CEX encounters as a workplace-based learning tool for final-year dental students: a convergent mixed-methods study
Source: BMC Med Educ. 2026 Jul 25;26:1694. doi: 10.1186/s12909-026-09955-5 (PMC13689196; doi:10.1186/s12909-026-09955-5)
Supplement: Supplementary file 1 [file 12909_2026_9955_MOESM1_ESM.docx]

**Supplementary File 1**

**Additional supporting tables for the Mini-CEX mixed-methods study**

This supplementary file provides additional methodological and analytical details that support the manuscript. It intentionally avoids duplicating the main Results tables. The supplementary material should be used for transparency, verification, and expanded detail.

**Supplementary Table S1. Normality testing of paired difference scores and primary test selection**

| **Outcome** | **SD of paired difference** | **Shapiro-Wilk p-value** | **Primary paired test** |
| --- | --- | --- | --- |
| Medical interviewing skills | 1.38 | 0.003 | Wilcoxon signed-rank test |
| Humanistic qualities/professionalism | 1.56 | 0.013 | Wilcoxon signed-rank test |
| Overall clinical competence | 1.56 | 0.010 | Wilcoxon signed-rank test |
| Student satisfaction | 0.96 | <0.001 | Wilcoxon signed-rank test |
| Observation time | 2.58 | <0.001 | Wilcoxon signed-rank test |
| Feedback time | 2.51 | <0.001 | Wilcoxon signed-rank test |

**Note.** Shapiro-Wilk p-values <0.05 indicate non-normal paired differences. Domains with zero paired differences were not inferentially tested.

**Supplementary Table S2. Extended paired comparison results with confidence intervals and sensitivity analysis**

| **Outcome** | **First encounter Mean ± SD** | **Second encounter Mean ± SD** | **Mean difference** | **95% CI for mean difference** | **Wilcoxon p-value** | **Paired t-test p-value** | **Cohen’s dz for paired t-test sensitivity analysis** |
| --- | --- | --- | --- | --- | --- | --- | --- |
| Medical interviewing skills | 4.54 ± 1.38 | 4.93 ± 0.52 | 0.39 | -0.04 to 0.82 | 0.047 | 0.077 | 0.28 |
| Humanistic qualities/professionalism | 4.49 ± 1.40 | 5.07 ± 0.69 | 0.59 | 0.09 to 1.08 | 0.019 | 0.021 | 0.37 |
| Overall clinical competence | 4.61 ± 1.39 | 5.00 ± 0.63 | 0.39 | -0.10 to 0.88 | 0.081 | 0.118 | 0.25 |
| Student satisfaction | 5.56 ± 0.63 | 7.24 ± 0.73 | 1.68 | 1.38 to 1.99 | <0.001 | <0.001 | 1.75 |
| Observation time | 21.71 ± 2.40 | 7.33 ± 0.94 | -14.38 | -15.19 to -13.56 | <0.001 | <0.001 | -5.58 |
| Feedback time | 14.39 ± 2.55 | 3.48 ± 0.73 | -10.91 | -11.70 to -10.11 | <0.001 | <0.001 | -4.34 |

**Note. The** Wilcoxon signed-rank test was used as the primary test. Paired t-tests and confidence intervals are provided as sensitivity/verification statistics.

**Supplementary Table S3. Distribution of Mini-CEX performance categories by encounter**

| **Domain** | **Encounter** | **Unsatisfactory n (%)** | **Satisfactory n (%)** | **Superior n (%)** |
| --- | --- | --- | --- | --- |
| Medical interviewing skills | First | 13 | 28 | 0 |
| Medical interviewing skills | Second | 1 | 40 | 0 |
| Physical examination skills | First | 2 | 39 | 0 |
| Physical examination skills | Second | 2 | 39 | 0 |
| Humanistic qualities/professionalism | First | 13 | 28 | 0 |
| Humanistic qualities/professionalism | Second | 0 | 40 | 1 |
| Clinical judgment | First | 2 | 39 | 0 |
| Clinical judgment | Second | 2 | 39 | 0 |
| Counseling skills | First | 1 | 40 | 0 |
| Counseling skills | Second | 1 | 40 | 0 |
| Organization/efficiency | First | 1 | 40 | 0 |
| Organization/efficiency | Second | 1 | 40 | 0 |
| Overall clinical competence | First | 13 | 28 | 0 |
| Overall clinical competence | Second | 1 | 40 | 0 |

**Note.** Performance bands were based on the Mini-CEX scale: 1-3 unsatisfactory, 4-6 satisfactory, and 7-9 superior.

**Supplementary Table S4. Expanded qualitative coding framework**

| **Analytical area** | **Subtheme** | **Example codes** |
| --- | --- | --- |
| Expectations and emotional anticipation | Anticipation; apprehension; curiosity; optimism | nervousness, curiosity, apprehension, excitement, pressure, opportunity, meaningful assessment |
| Preparation strategies | Self-directed preparation; peer/mentor feedback; practice | reviewed clinical guidelines, online resources, role-play, simulation exercises, clinical skills practice, communication rehearsal, feedback from peers and mentors |
| Emotional experience during Mini-CEX | Anxiety; confidence; motivation; emotional transition | anxiety, fear, nervousness, excitement, confidence, focus, motivation, hesitation, positive emotional experience |
| Feedback and learning value | Constructive feedback, actionable learning, and confidence building | real-time feedback, targeted skill development, valuable insights, identified areas for improvement, actionable insights, boosted confidence, improved competence |
| Faculty support and assessor behavior | Guidance; encouragement, variation in support | excellent support, helpful and encouraging, constructive feedback, useful guidance, non-judgmental attitude, supportive attitude, less comprehensive guidance, feeling unsupported |
| Standardization concerns | Variation in assessment conduct | variations, inconsistency, need for more standardized procedures, need for detailed guidance |
| Professional growth | Professional competency; holistic development; resilience | personal development, professional behaviors, resilience and growth, refined skills, holistic development, confidence in clinical role |

**Note.** The table summarizes expanded codes from the qualitative coding matrix. No patient-identifiable or student-identifiable information is included in the coding framework.
