# Supplementary material for: Repeated Mini-CEX encounters as a workplace-based learning tool for final-year dental students: a convergent mixed-methods study
Source: BMC Med Educ. 2026 Jul 25;26:1694. doi: 10.1186/s12909-026-09955-5 (PMC13689196; doi:10.1186/s12909-026-09955-5)
Supplement: Supplementary file 2 [file 12909_2026_9955_MOESM2_ESM.docx]

**Supplementary File 2. Reflective Essay Guide for the Qualitative Component**

*Study: Repeated Mini-CEX encounters as a workplace-based learning tool for final-year dental students*

# Purpose of the tool

This supplementary tool was used to collect qualitative data after completion of the second Mini-CEX encounter. The guide was designed to elicit students' reflections on emotional response, feedback experience, confidence, clinical learning, faculty support, standardization concerns, and perceived professional growth. The prompts are open-ended and are not intended for scoring or grading.

# Administration instructions

**•** Administer after the second Mini-CEX encounter so that students can reflect on both encounters.

**•** Allow students three working days to complete the reflection.

**•** Permit either handwritten or typed responses.

**•** Use participant codes instead of names or roll numbers before analysis.

**•** Inform students that participation is voluntary and that responses will not affect academic evaluation.

**•** Encourage honest reflection; there are no right or wrong answers.

# Instructions for students

Please write a reflective response about your experience of the Mini-CEX encounters. You may answer each prompt separately or write one continuous reflection. You do not need to include your name. Please write honestly about what helped your learning, what was difficult, and what could be improved. Your response will be anonymized before analysis and will not be used for academic grading. Please do not include patient names, patient identifiers, or any information that could identify a patient. Please avoid mentioning faculty names or any identifiable information about assessors.

# Reflective essay prompts

| **No.** | **Prompt for reflective essay** | **Analytic focus aligned with Results** |
| --- | --- | --- |
| 1 | Describe your initial feelings before or during the Mini-CEX encounter. What made you feel comfortable or uncomfortable? | Emotional response; anxiety; motivation; psychological safety |
| 2 | How did you prepare for the Mini-CEX encounters? Did your preparation change between the first and second encounter? | Preparation; self-directed learning; readiness for clinical encounter |
| 3 | Describe your experience of being directly observed while interacting with a patient. | Direct observation; real patient-based learning; performance under observation |
| 4 | What type of feedback did you receive after the encounter? Which parts of the feedback were most useful for your learning? | Feedback specificity; usefulness; actionable correction; feedback quality |
| 5 | Did the feedback influence your confidence or your approach to the next clinical encounter? Please explain. | Confidence-building; feedback uptake; application of feedback |
| 6 | In what ways did the Mini-CEX help you connect theoretical knowledge with clinical practice? | Theory-practice integration; experiential learning; clinical reasoning |
| 7 | Did the Mini-CEX help you improve any specific clinical skills, such as history-taking, communication, professionalism, counseling, or organization? | Perceived skill development; interviewing; professionalism; competence domains |
| 8 | How did the assessor's attitude, communication style, or support affect your learning experience? | Faculty support; psychological safety; assessor behavior; engagement |
| 9 | Were there any concerns about fairness, standardization, timing, or feedback quality during the Mini-CEX process? | Standardization concerns; feasibility; variation in guidance; feedback comprehensiveness |
| 10 | Overall, what did you learn from the Mini-CEX experience, and how might it influence your future role as a dentist? | Reflective learning; professional growth; identity formation; future practice |

# Optional probing questions

**•** Can you give an example from the encounter?

**•** What changed between your first and second Mini-CEX experience?

**•** What did the feedback help you notice about your own performance?

**•** What would make Mini-CEX more useful for future students?
